# Detection of prostate cancer using diffusion-relaxation correlation spectrum imaging with support vector machine model – a feasibility study

**DOI:** 10.1186/s40644-022-00516-9

**Published:** 2022-12-27

**Authors:** Xiaobin Wei, Li Zhu, Yanyan Zeng, Ke Xue, Yongming Dai, Jianrong Xu, Guiqin Liu, Fang Liu, Wei Xue, Dongmei Wu, Guangyu Wu

**Affiliations:** 1 Department of Radiology, Renji Hospital, School of Medicine, Shanghai Jiao Tong University, Shanghai, China; 2 Quanzhou Maternity and Children’s Hospital, Fujian, China; 3 Central Research Institute, MR Collaboration, United Imaging Healthcare, Shanghai, China; 4 Department of Urology, Renji Hospital, School of Medicine, Shanghai Jiao Tong University, Shanghai, China; 5 Shanghai Key Laboratory of Magnetic Resonance, School of Physics and Electronics Science, East China Normal University, Shanghai, China

## Abstract

**Background:**

To evaluate the performance of diffusion-relaxation correlation spectrum imaging (DR-CSI) with support vector machine (SVM) in detecting prostate cancer (PCa).

**Methods:**

In total, 114 patients (mean age, 66 years, range, 48–87 years) who received a prostate MRI and underwent biopsy were enrolled in three stages. Thirty-nine were assigned for the exploration stage to establish the model, 18 for the validation stage to choose the appropriate scale for mapping and 57 for the test stage to compare the diagnostic performance of the DR-CSI and PI-RADS.

**Results:**

In the exploration stage, the DR-CSI model was established and performed better than the ADC and T_2_ values (both *P* < 0.001). The validation result shows that at least 2 pixels were required for both the long-axis and short-axis in the mapping procedure. In the test stage, DR-CSI had higher accuracy than PI-RADS ≥ 3 as a positive finding based on patient (84.2% vs. 63.2%, *P* = 0.004) and lesion (78.8% vs. 57.6%, *P* = 0.001) as well as PI-RADS ≥ 4 on lesion (76.5% vs. 64.7%, *P* = 0.029), while there was no significant difference between DR-CSI and PI-RADS ≥ 4 based on patient (*P* = 0.508). For clinically significant PCa, DR-CSI had higher accuracy than PI-RADS ≥ 3 based on patients (84.2% vs. 63.2%, *P* = 0.004) and lesions (62.4% vs. 48.2%, *P* = 0.036). There was no significant difference between DR-CSI and PI-RADS ≥ 4 (*P* = 1.000 and 0.845 for the patient and lesion levels, respectively).

**Conclusions:**

DR-CSI combined with the SVM model may improve the diagnostic accuracy of PCa.

**Trial registration:**

This study was approved by the Ethics Committee of our institute (Approval No. KY2018-213). Written informed consent was obtained from all participants.

## Background

Prostate cancer (PCa) is the second most frequently occurring cancer among males, with the highest incidence rates in over 60% countries worldwide, and remains a leading cause of cancer-related death in many countries, imposing a heavy burden on families and communities [1]. The accurate detection of PCa at an earlier stage is of vital importance for clinical decision-making, the evaluation of long-term survival and better outcomes/prognosis. For clinical prostate examination, magnetic resonance imaging (MRI) is a significant imaging modality that provides superb soft tissue contrast and functional evaluation. To date, multiparametric MRI (MP-MRI), including T_2_-weighted imaging (T2WI), diffusion-weighted imaging (DWI), and dynamic contrast-enhanced MRI (DCE-MRI), has been proven to be a promising noninvasive tool for PCa detection [2–4].

Among the metrics derived from MP-MRI, T_2_ and apparent diffusion coefficient (ADC) are widely accepted as the two most valuable biomarkers for tumour characterization [4, 5]. Furthermore, both T_2_ and ADC have been found to be related to the aggressiveness of PCa, which is indicated by the Gleason score [6, 7]. However, with the increasing application of MP-MRI using the Prostate Imaging-Reporting and Data System Version 2.1 (PI-RADS v2.1) for PCa diagnosis, it was found that approximately 15–30% of clinically significant cancers were undetected, which, to some extent, depends on the clinical experience of the radiologists [8, 9]. As such, accurate PCa diagnosis remains a challenge [10–12].

Concerning PCa, multiple intravoxel components with different or similar MR properties, such as T_2_ or ADC, add difficulty for MP-MRI in differentiation. In addition, various tissue structural features at a microscopic scale produce another obstacle for visualization owing to limited MRI spatial resolution. To resolve this issue, some attempts have been made in multidimensional MRI, a method that tries to disentangle intravoxel signals in terms of parametric spectra, such as T_1_, T_2_ and ADC, and hence to infer the intravoxel tissue composition [13, 14]. For example, hybrid multidimensional MRI (HM-MRI) was used to probe the stroma, epithelium, and lumen changes in PCa [14, 15]. Moreover, the novel diffusion-relaxation correlation spectrum imaging (DR-CSI) method was proposed [16] and successfully identified and quantified multiple distinct components (epithelium, stroma, and lumen) in the ex vivo prostate through a T_2_–ADC spectrum with prior knowledge [17]. Owing to the heterogeneous tissue structure and biological environment in vivo, it is difficult to know the prior knowledge to make a tissue structural assumption about PCa based on which DR-CSI could apply. Nevertheless, to our knowledge, no research on DR-CSI for in vivo PCa detection has been initiated yet.

In this study, the machine learning technique, the support vector machine (SVM) model, was introduced in combination with DR-CSI. SVM, as a supervised ML technique that tries to find the optimal hyperplane that maximizes the margin between two classes, has been widely used for the detection and classification of PCa due to its mature mathematical formulation, flexibility, high accuracy, robust theoretical support, direct geometric interpretation, and wide availability of software implementations [18–23]. For example, integrating with the parameters of MP-MRI, such as diffusion and perfusion, SVM enabled accurate and automatic classification of low-grade and high-grade PCa in the central gland [23]. Combined with DR-CSI, the SVM model was used to analyse spectral results according to the PCa diagnosis results instead of direct biologic interpretation based on a tissue structural assumption. The purpose of this study was to evaluate the feasibility of DR-CSI combined with an SVM model for the detection of PCa in vivo and to compare its diagnostic performance with PI-RADS v2.1 scores.

## Methods

### Patients

This retrospective study was approved by the Ethics Committee of our institute (Approval No. KY2018-213). Written informed consent was obtained from all participants. Three hundred and twenty consecutive patients who were scheduled for prostate MRI were recruited for this study between August 2020 and December 2021. The inclusion criteria were as follows: (a) prostate-specific antigen (PSA) elevated to ≥ 4 ng/mL or digital rectal examination (DRE) positive; (b) complete MRI examination and clinical data; and (c) no surgery before MRI examination. The exclusion criteria were as follows: (a) images with poor quality or artefacts; (b) patients with a previous history of prostate biopsy, prostate surgery, or other treatment; and (c) time interval between MRI and biopsy ≥ 3 months. In this study, 12 patients with previous chemotherapy, 5 patients with previous operation or treatment for PCa, and 2 patients with poor quality or artefacts were excluded. Three hundred and one patients were included (100 for the exploration stage, 56 for the validation stage and 145 for the test stage). In total, 114 patients who underwent biopsy were enrolled in three stages. Detailed participant characteristics are summarized in Table 1.

**Table 1:** Patient characteristics

Variable	Total	Exploration stage	Validation stage	Test stage
No. of patients	301	100	56	145
Mean age (y)	67	67	66	67
Mean PSA (ng/mL)^a^	6.98	6.29	9.52	6.47
No. of patients with biopsy	114	39	18	57
No. of patients with benign results	48	17	6	25
No. of patients with PCa	66	22	12	32

^a^*PSA* Prostate-Specific Antigen, *PCa* prostate cancer

### Study design

**Fig. 1:**
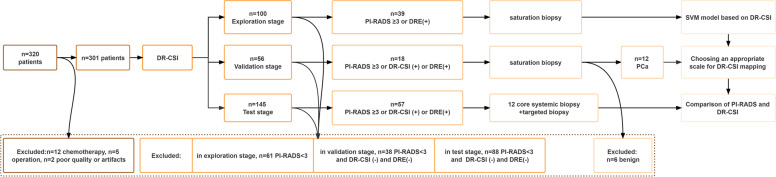
Flowchart of the study design. DR-CSI, diffusion-relaxation correlation spectrum imaging; SVM, support vector machine; DRE, digital rectal examination; PI-RADS, Prostate Imaging—Reporting and Data System

After prostate MRI, patients were enrolled in three stages – the exploration, validation, and test stages. In the exploration stage, patients with PI-RADS ≥ 3 lesions or DRE positivity underwent biopsy, and a PCa detection model utilizing an SVM model was established based on the DR-CSI and biopsy results. Then, in the validation stage, an optimal filter scale for the SVM model was chosen with the images used to detect PCa from biopsy, in which the biopsies were carried out on patients with PI-RADS ≥ 3 lesions or positive results according to the PCa detection model or DRE positivity. Finally, in the test stage, the PCa detection model was used to predict PCa, and the results were compared with PI-RADS scores as well as the gold standard. The biopsy results of patients had the same criteria as in the validation stage. The comparison was performed based on patient level as well as lesion level. At the lesion level, a single lesion was defined as a positive targeted lesion in DR-CSI or PI-RADS. PCa proven in 12 systemic biopsies with negative imaging results was also defined as a single lesion. For patients biopsied due to positive DRE results with negative imaging and pathology results, the whole prostate was defined as a single case. The flowchart of the study design is presented in Fig. 1.

### MR Imaging acquisition

All MRI examinations were performed on a 3.0 T scanner (uMR 780, United Imaging Healthcare, Shanghai, China) with a commercial body phased array coil. MR examination included T2WI, DWI, DCE-MRI, T_1_-weighted imaging (T1WI) and DR-CSI. T1WI and T2WI were acquired. The detailed MR protocols are presented in Table 2. DR-CSI was also performed based on the SS SE-EPI including a matrix of 6*6 data points of echo times (57, 75, 100, 120, 150, 180 ms) and b-values (0, 150, 400, 800, 1200, 1500 s/mm^2^). The SS SE-EPI sequence was performed 6 times for each patient. For each time, TE was set as a specific value in its range with a full set of b-values (0, 150, 400, 800, 1200, 1500 s/mm^2^), i.e., diffusion sequence with different T_2_ weights. As TE was increased, diffusion time was increased in parallel.

**Table 2:** Scanner parameters of MR sequences

Sequences	DR-CSI	Axial FSE T1W	Coronal FSE T1W	Axial FSE T2W	Coronal FSE T2W
Parameters					
TR (ms)	3000	600	630	4792	4000
TE (ms)	57 (min-value)	9.22	8.86	149.04	141.84
FA (deg)	90	90	90	90	90
FOV (cm^2^)	20 × 20	20 × 20	22 × 22	18 × 18	22 × 22
Matrix	112 × 112	256 × 192	288 × 216	240 × 240	304 × 304
Intersection Gap (mm)	0.35	0.30	0.30	0	0
Slice thickness (mm)	3.5	3	3	3	3
Slices	5–15	20	20	28	24
Acquisition time	7 min 30 s/per 5 slices	2 min 16 s	2 min 26 s	3 min 02 s	2 min 16 s

^a^*DR-CSI* diffusion-relaxation correlation spectrum imaging, *FSE* fast spin echo, *TR* repetition time, *TE* echo time, *FA* flip angle, *FOV* field of view

### DR-CSI Postprocessing

According to DR-CSI theory [16], the MR signal is expressed as an integral of exponential decay functions characterized by T_2_ and D:

1 $$S(x,y,TE,b)=\iint w(x,y,T2,D){{e}^{-TE/T2}e}^{-b\bullet D}$$

where T_2_ and D represent the transverse relaxation time and the diffusivity, respectively. $\mathrm{w}(\mathrm{x},\mathrm{y},\mathrm{T}2,\mathrm{D})$ stands for a 4D distribution function that connects the T_2_-D spectrum with each spatial location.

In practice, the signal needs to be expressed in a discretized form:

2 $$S\left(x,y,TE,b\right)=\sum_{j=1}^{J}\sum_{i=1}^{I}{w}_{i,j}(x,y,T{2}_{i},{D}_{j}){e}^{-TE/T{2}_{i}}{e}^{-b{\bullet D}_{j}}$$

where I and J are the numbers of T_2_ and D, respectively. T2_i_ and D_j_ represent the transverse relaxivity and diffusivity, respectively. ${\mathrm{w}}_{\mathrm{i},\mathrm{j}}(\mathrm{x},\mathrm{y},\mathrm{T}{2}_{\mathrm{i}},{\mathrm{D}}_{\mathrm{j}})$ is a discrete 4D distribution function described above.

The distribution function was obtained through spectrum estimation, i.e., finding the solution to the above equation with consistency, a nonnegativity and a spatial smoothness constraint following steps in a previous study [16].

To analyse component distributions in regions of interest (ROIs), the T_2_-D spectra space was empirically segmented into six subregions (i.e., components). A previous study demonstrated that the epithelial structure was more likely to be present within the scale of T_2_ < 50 ms and D < 0.5 mm^2^/μs based on ex vivo imaging [18]. Considering the enrichment of epithelial structure in PCa [14, 15], these two values were selected as the cut-offs for further classification. Moreover, D = 3 mm^2^/μs was also adopted as the cut-off value because the diffusion velocity of water molecules beyond 3 mm^2^/μs may likely characterize particular physiological information (e.g., perfusion, secretion). Thus, the ranges of the subregions were finally classified as follows: 1) T_2_ < 50 ms, D < 0.5 mm^2^/μs; 2) T_2_ > 50 ms, D < 0.5 mm^2^/μs; 3) T_2_ < 50 ms, 0.5 mm^2^/μs < D < 3 mm^2^/μs; 4) T_2_ > 50 ms, 0.5 mm^2^/μs < D < 3 mm^2^/μs; 5) T_2_ < 50 ms, D > 3 mm^2^/μs; and 6) T_2_ > 50 ms, D > 3 mm^2^/μs. The corresponding fraction of each subregion, (${f}_{i}, i=\mathrm{1,2},\dots 6$), for each voxel was estimated by the normalization of distribution functions:

3 $${f}_{i}\left(x,y\right)=\frac{\sum_{{T}_{2},D \in {subregion}_{i}}w(x, y, {T}_{2},D)}{\sum_{{T}_{2},D \in whole spectra}w(x, y, {T}_{2},D)}, i\in \left[\mathrm{1,6}\right]\cap {\mathbb{Z}}$$

where $i$ denotes the order of the segmented subregion of the T_2_-D spectral space.

### Image analysis

**Fig. 2:**
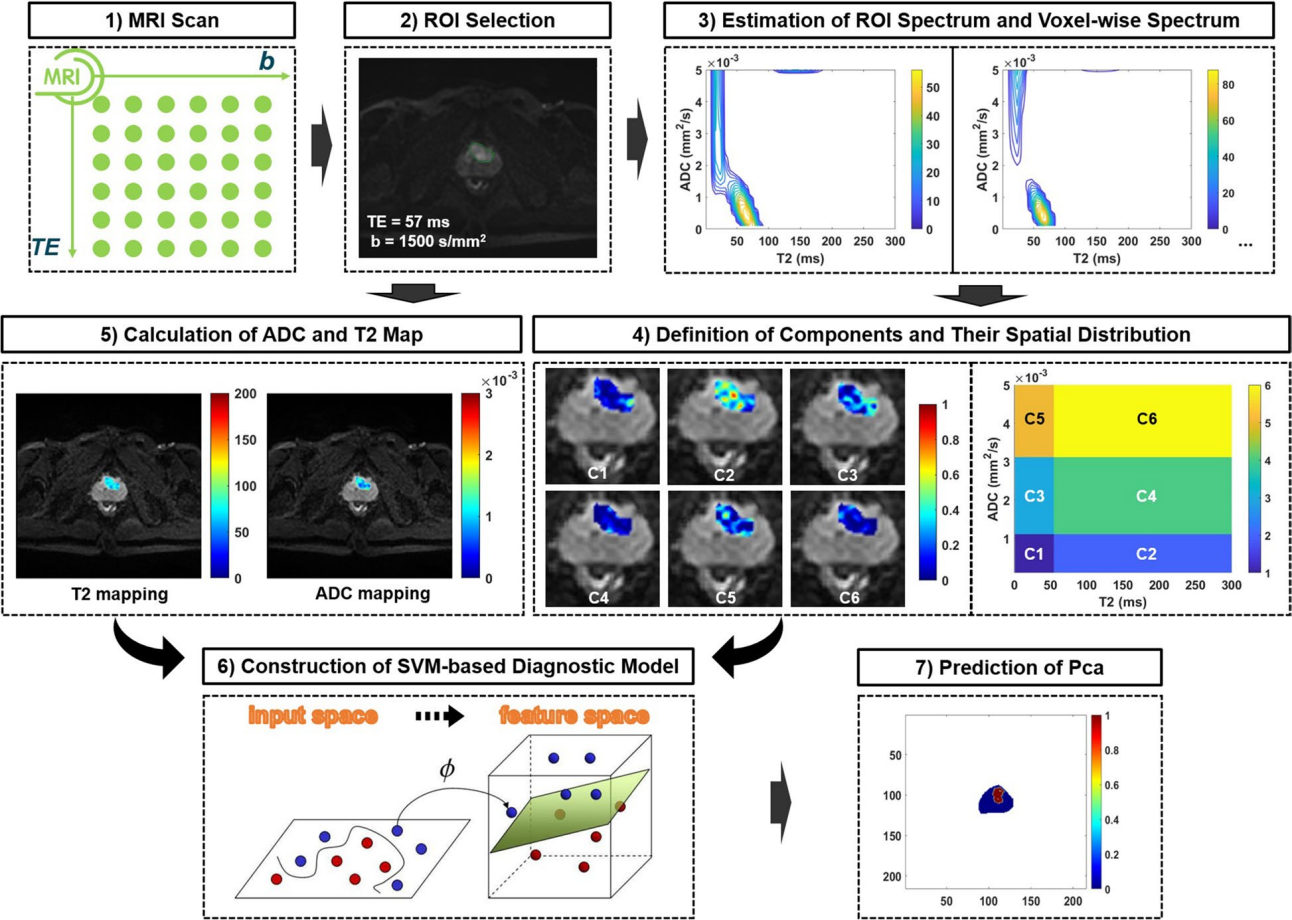
Illustration of the processes. Illustration of the processes for diffusion-relaxation correlation spectrum imaging (DR-CSI), support vector machine (SVM) model construction, and cancer detection performance

**Fig. 3:**
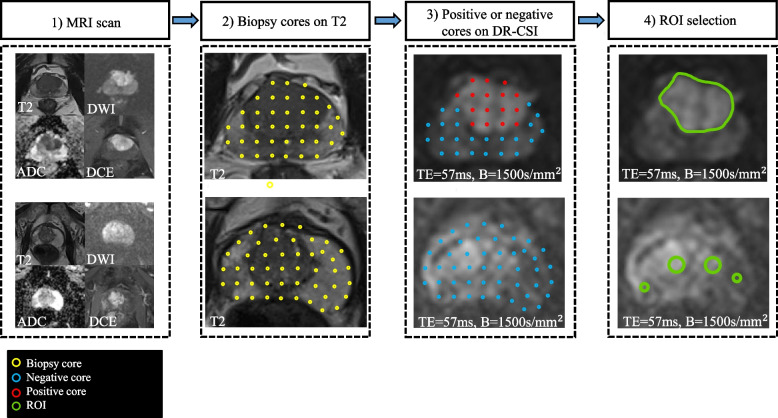
The process of labelling the ROI. The first row shows a biopsy-positive patient with PI-RADS 5. The second row shows a biopsy-negative patient with PI-RADS 2 but positive in DRE

Figure 2 presents the whole process of image analysis. All image analyses were processed using MATLAB R2021a software (MathWorks, Natick, MA, USA). In the exploration and validation stage, the saturation prostate biopsy scheme was performed with US-MRI fusion biopsy (Esaote, Genoa, Italy). The median number of biopsy cores was 23. The location of the biopsy was further remarked on MRI [24]. Then, an experienced radiologist (G. W with 14 years of experience in prostate MR imaging) drew the ROI presented with PI-RADs ≥ 3 within 3 mm from the positive core of biopsies based on DW image with b-value = 1500 s/mm^2^ with the lowest TE (57 ms) [25–27]. For the cases with a negative biopsy result, circular ROIs with a radius from 3 to 8 mm were delineated on the bilateral central and peripheral areas in the largest area of the prostate (Fig. 3). Patients in the test stage underwent 12 core systemic biopsies, combined with targeted biopsies from MP-MRI and DR-CSI, which we designed to assess, with each core identified and processed separately. The ROIs of the whole prostate were delineated in the test stages. ROI drawn by another radiologist (with 11 years of experience in prostate MR imaging) was used to assess the interobserver agreement of the technique.

Accordingly, the fraction maps corresponding to six subregions of each ROI were acquired through DR-CSI analysis. Meanwhile, conventional voxelwise T_2_ and ADC maps of all ROIs were estimated following a monoexponential signal decay model using part of the DR-CSI data points (b = 0, TE = 57/75/100/120/150/180 ms; TE = 57 ms, b = 0/800 s/mm^2^), respectively [7].

The SVM-related procedures were as follows. First, the quantitative imaging features (T_2_, ADC and six component fractions) of all voxels from all patients in the exploration stage were used for the construction of the PCa detection model. Then, an SVM classifier with a linear kernel for PCa prediction was trained and established through tenfold cross validation, which evenly split the dataset into 10 subsets with 9 subsets for training and the last subset for evaluation. This process was repeated 10 times, leaving one different subset for evaluation each time. Second, different filter scales with different numbers of pixels (i.e., 1*1, 1*2, 1*3, …, 5*5) were used in the validation stage to choose an optimal scale, which could reduce the false-positive results resulting from the SVM-based PCa detection model. Then, the optimal filter scale with the highest Dice ratio between the modelling results and biopsy results was determined. Third, the SVM-based PCa detection model with the optimal filter scale was used in the test stage, and the voxelwise model-predicted mapping results were generated.

The PI-RADS score was determined by an experienced radiologist (with more than 10 years of experience in prostate MR imaging) unaware of the biopsy results in the exploration and validation stage. In the test stage, the GS of the lesions was assigned according to the most common GS in the biopsy result.

### Statistical analysis

Statistical analysis was performed by SPSS 24.0 (IBM Corp, NY, USA). Kappa values < 0.20 indicated slight agreement, values of 0.21–0.40 indicated fair agreement, values of 0.41–0.60 indicated moderate agreement, values of 0.61–0.80 indicated substantial agreement, and values > 0.81 indicated almost perfect agreement [28]. In the exploration stage, the performance of DR-CSI was compared with traditional ADC and T_2_ maps in differentiating cancer from non-PCa tissue at the largest Youden index point in receiver operating characteristic (ROC) analysis based on each voxel. In the test stage, the comparison of accuracy between DR-CSI and PI-RADS in terms of the diagnosis of PCa or cs-PCa was assessed by the McNemar test. Differences were considered significant when the *P* value was < 0.05.

## Results

**Fig. 4:**
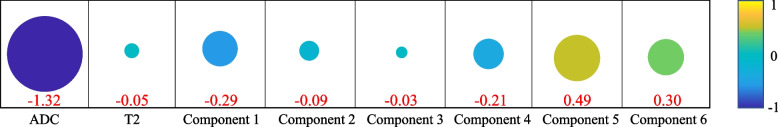
Weight map. The weight map demonstrated the weight of the apparent diffusion coefficient (ADC), T_2_ value, and six signal component fractions’ contribution to the SVM model

**Fig. 5:**
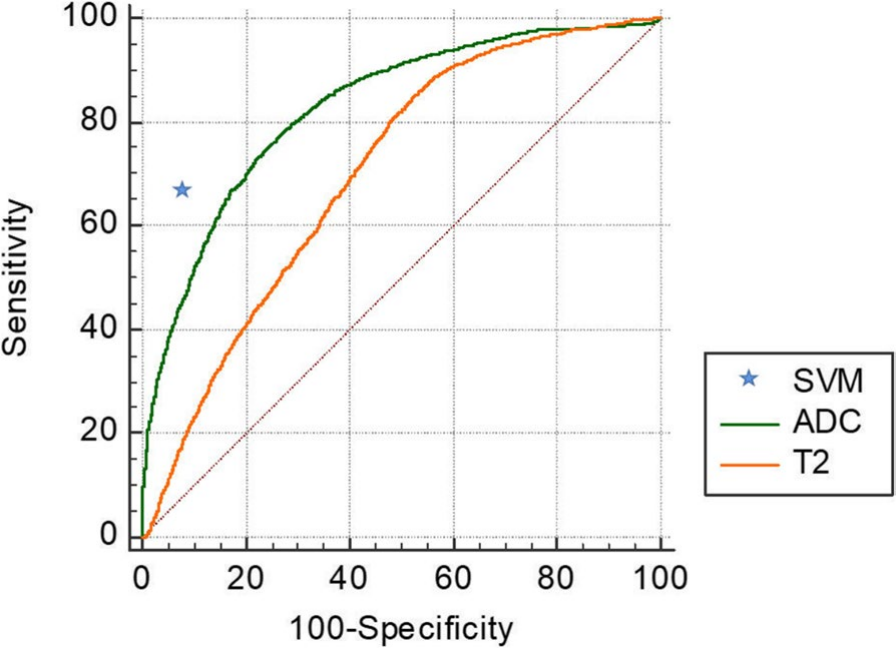
Diagnostic performance. Diagnostic performance of the support vector machine (SVM) model, apparent diffusion coefficient (ADC), and T_2_ value

In the exploration stage, a total of 39 patients underwent biopsy, among which 3 were PI-RADS 2 but DRE positive, 21 were PI-RADS 3, 10 were PI-RADS 4, and 5 were PI-RADS 5. There were 72 lesions (4 lesions with PI-RADS 2, 31 lesions with PI-RADS 3, 27 with PI-RADS 4, and 10 with PI-RADS 5). The maximal diameter of the index lesion ranged from 0.4–3.9 cm (median = 1.3 cm). Of these, 17 patients were confirmed to have benign disease (3 with PI-RADS 2, 10 with PI-RADS 3, 4 with PI-RADS 4), and 22 patients had PCa (11 with PI-RADS 3, 6 with PI-RADS 4, 5 with PI-RADS 5). There were 29 PCa lesions (12 lesions with PI-RADS 3, 9 with PI-RADS 4, and 8 with PI-RADS 5). A total of 10,434 tumour voxels (3453 with Gleason grade = 6, 3875 with Gleason grade ≥ 7, and 3106 non-PCa tissue voxels) were included in the analysis to establish an SVM model. Figure 4 shows the weights of T_2_ and ADC values and six subregions contributing to the SVM model. Figure 5 shows the diagnostic performance of the DR-CSI model, traditional ADC and T_2_ value. The DR-CSI model was more accurate than the traditional ADC (0.87 vs. 0.81, *P* < 0.001) and T_2_ value (0.87 vs. 0.70, *P* < 0.001) at the highest Youden index point. Interreader agreement analysis showed that almost perfect agreement was achieved in each subregion (kappa value = 0.99, 1.00, 0.98, 0.99, 0.99 and 0.98 for components 1–6, respectively).

**Fig. 6:**
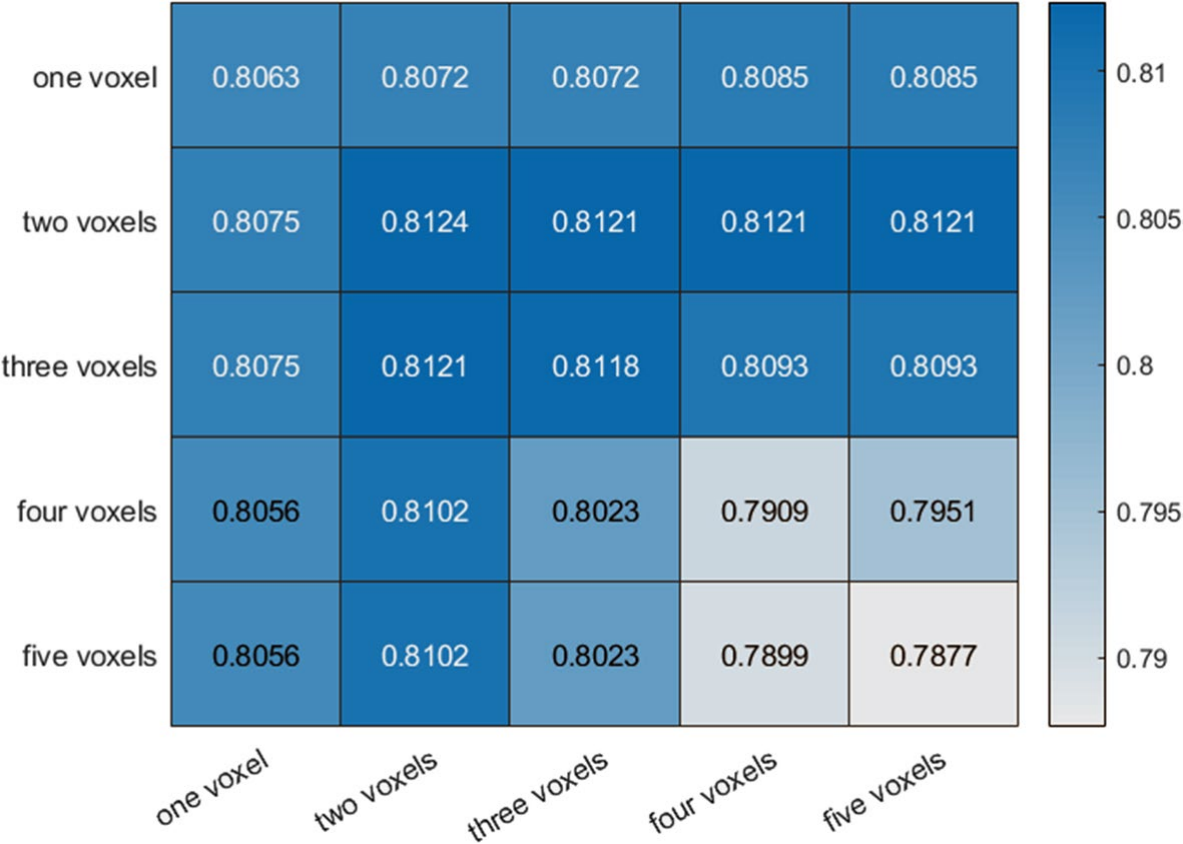
Dice ratio with different mapping voxel scales. Dice ratio calculated in diffusion-relaxation correlation spectrum imaging (DR-CSI) with different mapping voxel scales

In the validation stage, a total of 18 patients (17 patients with PI-RADS ≥ 3 or DR-CSI positivity and 1 patient with PI-RADS < 3 and DR-CSI negativity but DRE positivity) underwent biopsy. Of these, 6 patients were confirmed to have benign disease, and 12 had PCa. We chose those 12 images and compared the performance of different mapping methods to choose an appropriate scale for further testing. Among each scale, the largest Dice ratio was found in the scale, with the long axis and the short axis having at least 2 and 2 pixels, respectively (Fig. 6). The scale was further applied in the test stage.

**Fig. 7:**
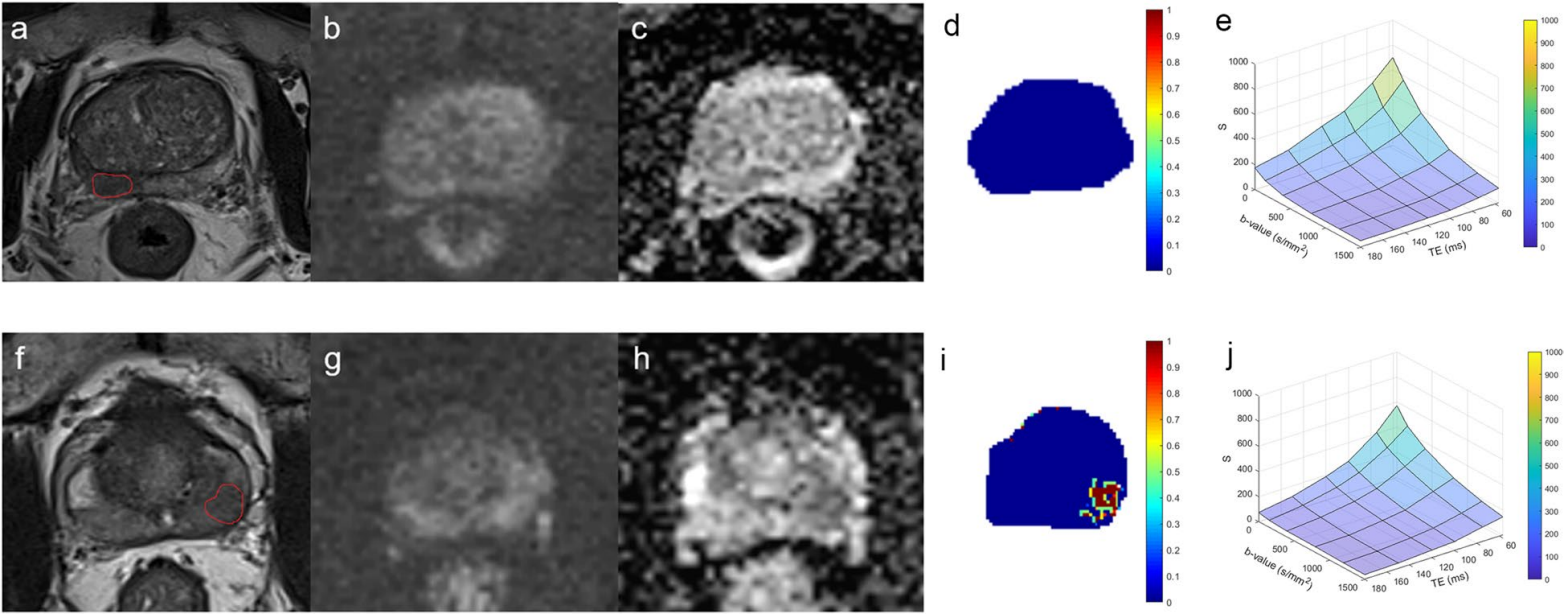
Examples of lesion detection. The first row shows a 74-year-old patient, tPSA 5.97 ng/mL, diagnosed with PI-RADS 3 at the right middle-anterior peripheral zone but negative in DR-CSI, who was finally confirmed to be benign by biopsy. The second row shows a 65-year-old patient, tPSA 7.79 ng/mL, diagnosed with PI-RADS 2 but positive in DR-CSI in the left middle peripheral zone, who was finally confirmed to have PCa and a Gleason score of 3 + 3 by biopsy. (**a** and **f**) T2WI; (**b** and **g**) diffusion weighted imaging; (**c** and **h**) apparent diffusion coefficient; (**d** and **i**) diffusion-relaxation correlation spectrum imaging (DR-CSI) result; (**e** and **j**) Decreasing of signal intensity in lesions with PI-RADs 3 delineated in the DR-CSI images (**a **& **f**)

In the test stage, a total of 57 patients underwent biopsy, among which 7 patients were PI-RADS < 3 and DR-CSI negative but DRE positive, 1 was PI-RADS 2 but DR-CSI positive, 28 were PI-RADS 3, 13 were PI-RADS 4, and 8 were PI-RADS 5. The maximal diameter of the index lesion ranged from 0.3–3.2 cm with a median of 1.1 cm. Of these, 25 patients were confirmed to have benign disease (7 with PI-RADS 2, 11 with PI-RADS 3, 7 with PI-RADS 4), and 32 had PCa (1 with PI-RADS 2, 17 with PI-RADS 3, 6 with PI-RADS 4, 8 with PI-RADS 5). Eighty-five lesions were subsequently verified. Thirty-one were confirmed to be benign, and 54 were PCa. Two typical cases are represented in Fig. 7. We analysed separately based on patient and lesion (Table 3). Considering PI-RADS ≥ 3 as a positive finding, DR-CSI had higher accuracy than PI-RADS ≥ 3 based on patients (84.2% vs. 63.2%, *P* = 0.004) and lesions (78.8% vs. 57.6%, *P* = 0.001). Considering PI-RADS ≥ 4 as a positive finding, DR-CSI had higher accuracy than PI-RADS ≥ 4 based on lesions (76.5% vs. 64.7%, *P* = 0.029), while no significant difference was found based on patients (84.2% vs. 78.9%, *P* = 0.508). For the analysis of clinically significant PCa, considering PI-RADS ≥ 3 as a positive finding, DR-CSI had higher accuracy than PI-RADS ≥ 3 based on patients (84.2% vs. 63.2%, *P* = 0.004) and lesions (62.4% vs. 48.2%, *P* = 0.036). Considering PI-RADS ≥ 4 as a positive finding, there was no significant difference between DR-CSI and PI-RADS ≥ 4 based on patients (84.2% vs. 82.5%, *P* = 1.000) and lesions (62.5% vs. 64.7%, *P* = 0.845).

**Table 3:** Performance in the diagnosis of PCa using PI-RADS and DR-CSI

	Patient			Lesion		
	PI-RADS	DR-CSI	*p* value	PI-RADS	DR-CSI	*p* value
**Gleason Score = 6 and ≥ 7**						
PI-RADS ≥ 3						
sensitivity	31/32	31/32		42/54	48/54	
specificity	5/25	17/25		7/31	19/31	
accuracy	36/57(63.2%)	48/57(84.2%)	0.004	49/85(57.6%)	67/85(78.8%)	0.001
PI-RADS ≥ 4						
sensitivity	28/32	31/32		34/54	48/54	
specificity	17/25	17/25		21/31	19/31	
accuracy	45/57(78.9%)	48/57(84.2%)	0.508	55/85(64.7%)	67/85(76.5%)	0.029
**Gleason Score ≥ 7**						
PI-RADS ≥ 3						
sensitivity	30/30	30/30		27/32	30/32	
specificity	6/27	18/27		14/53	23/53	
accuracy	36/57(63.2%)	48/57(84.2%)	0.004	41/85(48.2%)	53/85(62.4%)	0.036
PI-RADS ≥ 4						
sensitivity	28/30	30/30		23/32	30/32	
specificity	19/27	18/27		32/53	23/53	
accuracy	47/57(82.5%)	48/57(84.2%)	1.000	55/85(64.7%)	53/85(62.4%)	0.845

^a^*PCa* prostate cancer, *PI-RADS* Prostate Imaging-Reporting and Data System Version, *DR-CSI* Diffusion-Relaxation Correlation Spectrum Imaging

## Discussion

In this study, the feasibility of DR-CSI combined with an SVM model for detecting PCa in vivo was initially explored, and its diagnostic performance was evaluated and compared with the PI-RADS score based on MP-MRI. The optimal performance of the former method suggested additional clinical value and potential to improve the detection of PCa, especially based on lesion level. The results demonstrated that DR-CSI could provide additional information for PCa characterization in some clinical scenarios (e.g., targeted biopsy).

Conventionally, T_2_ and ADC derived from MP-MRI independently have been widely used for cancer detection and have proven to be of clinical significance [29, 30]. Some studies have reported that a strong interdependence exists between the measured ADC and T_2_ and varies depending on the tissue composition in PCa [31, 32]. Conventional MRI methods, which only provide voxel-averaged information on a macroscopic level, cannot disentangle intravoxel heterogeneity reflected by water diffusivity and relaxivity. In this sense, the ability of conventional MRI methods to investigate the tissue structure and composition in tumours is limited. In our study, compared with T_2_ and ADC maps alone, the diagnostic accuracy was improved by the DR-CSI with the SVM model. This could be attributed to the fact that DR-CSI could resolve the distribution of tissue components according to different spectral regions, providing additional information on tissue composition or structural features at a subvoxel level and hence improving the clinical diagnosis of PCa.

With decades of efforts, multidimensional MRI has been used to infer intravoxel heterogeneity in tissue due to its outstanding power to resolve signals from distinct tissue components. In pioneering work, DR-CSI has shown the potential to discriminate spatially overlapping phantoms and separate white and grey matter in both normal and injured animal spinal cords [16]. Specifically, with a priori knowledge, a hybrid multidimensional MRI model was set up based on an assumption of three different components in PCa [14, 15]. Owing to the intravoxel heterogeneity of tissue structure and the presence of multiple components in PCa (such as stroma, epithelium, and lumen, but not limited to these components) [33], the simplified DR-CSI model may limit the evaluation of microstructure complexity. Recently, some studies explored and validated the relations between the histological examination results and signal component fractions derived from DR-CSI in ex vivo PCa and in vivo PCa [15, 17].

However, the clinical diagnosis of PCa by DR-CSI still needs further study. Particularly, it is worth noting that there still exist other challenges to DR-CSI application for the clinical diagnosis of PCa [16]. First, the interpretation of the spectral results, i.e., associating the spectral peaks with specific tissue components or microstructure features, is not straightforward. Second, it is challenging to identify and determine the boundaries of spectral peaks, as the peaks would overlap and merge with one another, especially in malignant tumours with high intravoxel heterogeneity, making it difficult to robustly measure and map compartmental signal fractions.

Instead of assigning an individual spectral peak in the T_2_-D spectrum to a specific component, dividing the entire T_2_-D spectral space into six areas with different ranges of T_2_ and D could be a feasible way to reflect the diffusion-relaxation correlation information and avoid the need to identify specific components in the high intravoxel heterogeneous tissue structure. Thus, our DR-CSI was established based on segmented spectral areas to evaluate tissue composition in the diagnosis of PCa rather than to define a specific peak.

As the SVM model is an efficient way to deal with classification problems [22], it was adopted to build a voxel-based classification model for PCa diagnosis based on the resulting T_2_-D spectrum without defining and explaining the associations between segmented spectral areas with multiple components in PCa on the premise of no adequate prior knowledge about the microstructure compositions in vivo PCa. The results of the training model easily converged and could be quickly translated into practical applications. Moreover, the SVM model can explore the importance of a specific factor with the weight of each component in the model. ADC was still an important factor in PCa diagnosis, while different compartments derived from DR-CSI can simultaneously contribute to the classification model. Interestingly, components 5 and 6, which were considered to be associated with tissue perfusion in vivo with D > 3 mm^2^/μs, also accounted for significant proportions in the model. This finding verified the importance of perfusion emphasized by PI-RADS [10, 12]. Moreover, this result demonstrated that it is of great value to include the information available only in in vivo DR-CSI to improve the practical performance.

In the validation stage, we used 2*2 voxels as the filters to establish the mapping model. It is important to choose an appropriate filter scale for image mapping in SVM. Since the EPI sequence on the imaging level in vivo is easily disturbed by various artefacts, the direct mapping method without adding filters may not reach satisfactory results, such as increased false-positive results. Lesions with 2*2 voxels will obviously provide more reliable information than lesions with a single voxel. The scale with 2*2 voxels is smaller than most prostate tumours that can be found by MRI and will not lead to missed diagnosis due to the choice of boundary.

In this study, we also evaluated the performance of the DR-CSI model in detecting cs-PCa. Although the performance of the DR-CSI model did not exceed that of PI-RADS, it still has some practical significance. As the result of the DR-CSI model can be automatically derived with SVM, the method also provides a potential idea to solve the problem of lacking reproducibility with PI-RADS in diagnosis [6]. Meanwhile, as the model was established to differentiate cancer lesions from benign lesions, the model certainly lacks high precision to distinguish between cc-PCa and cs-PCa. The performance in detecting cs-PCa may further improve if only lesions with GS ≥ 7 are used in modelling.

Our study had some limitations to be acknowledged. First, considering that the diagnostic performance of saturation biopsy was consistent with that of radical prostatectomy pathology in general [25, 34], saturation biopsy was adopted instead of whole-mount results in our study. However, the method can still omit some information on heterogeneity, the performance of the model might be improved with reference to the whole-mount result, and further study is needed to probe the issue. Second, although improved spectral sensitivity and resolution are expected from more data points, an increase in acquisition time cost is inevitable. Further research is needed to find the optimal choice of TE and b-values. Third, the impact of the DR-CSI protocol, for instance, the spatial resolution and SNR, on the spectral sensitivity and resolution of DR-CSI was not evaluated. Fourth, in this study, we adopt a method to divide the spectrum based on a priori knowledge, which is just one of many possible patterns. Further studies are needed to explore the optimal method of dividing the subareas of the spectrum. Investigation of the optimized DR-CSI protocol is warranted in future studies.

## Conclusions

In conclusion, the DR-CSI combined with the SVM model has the potential to improve the diagnostic accuracy of prostate cancer.

## Data Availability

The datasets used and analysed during the current study are available from the corresponding author on reasonable request.

## Abbreviations

DR-CSI: Diffusion-relaxation correlation spectrum imaging
SVM: Support vector machine
PCa: Prostate cancer
PI-RADS: Prostate Imaging-Reporting and Data System
MRI: Magnetic resonance imaging
MP-MRI: Multiparametric MRI
T2WI: T_2_-weighted imaging
T1WI: T_1_-weighted imaging
DWI: Diffusion-weighted imaging
DCE-MRI: Dynamic contrast-enhanced MRI
ADC: Apparent diffusion coefficient
HM-MRI: Hybrid multidimensional MRI
PSA: Prostate-specific antigen
DRE: Digital rectal examination
ROI: Region of interest
ROC: Receiver operating characteristic
